# Magnitude of Stratification in Human Populations and Impacts on Genome Wide Association Studies

**DOI:** 10.1371/journal.pone.0008695

**Published:** 2010-01-13

**Authors:** Ke Hao, Eugene Chudin, Danielle Greenawalt, Eric E. Schadt

**Affiliations:** Genetics Department, Rosetta Inpharmatics, a Wholly Owned Subsidiary of Merck & Co. Inc., Seattle, Washington, United States of America; University of Michigan, United States of America

## Abstract

Genome-wide association studies (GWAS) may be biased by population stratification (PS). We conducted empirical quantification of the magnitude of PS among human populations and its impact on GWAS. Liver tissues were collected from 979, 59 and 49 Caucasian Americans (CA), African Americans (AA) and Hispanic Americans (HA), respectively, and genotyped using Illumina650Y (Ilmn650Y) arrays. RNA was also isolated and hybridized to Agilent whole-genome gene expression arrays. We propose a new method (i.e., hgdp-eigen) for detecting PS by projecting genotype vectors for each sample to the eigenvector space defined by the Human Genetic Diversity Panel (HGDP). Further, we conducted GWAS to map expression quantitative trait loci (eQTL) for the ∼40,000 liver gene expression traits monitored by the Agilent arrays. HGDP-eigen performed similarly to the conventional self-eigen methods in capturing PS. However, leveraging the HGDP offered a significant advantage in revealing the origins, directions and magnitude of PS. Adjusting for eigenvectors had minor impacts on eQTL detection rates in CA. In contrast, for AA and HA, adjustment dramatically reduced association findings. At an FDR = 10%, we identified 65 eQTLs in AA with the unadjusted analysis, but only 18 eQTLs after the eigenvector adjustment. Strikingly, 55 out of the 65 unadjusted AA eQTLs were validated in CA, indicating that the adjustment procedure significantly reduced GWAS power. A number of the 55 AA eQTLs validated in CA overlapped with published disease associated SNPs. For example, rs646776 and rs10903129 have previously been associated with lipid levels and coronary heart disease risk, however, the rs10903129 eQTL was missed in the eigenvector adjusted analysis.

## Introduction

Genome-wide association studies (GWAS) have emerged as an important approach to identify common polymorphisms underlying complex traits. Allele frequency disparity due to systematic ancestry differences, otherwise known as population stratification (PS), can bias testing results and lead to artifactual associations, although there is not yet consensus on how significant such a bias could be. Two general strategies were developed to address the PS risk. First, family-based design is robust against PS [1], [2]. These methods are effective but require first-degree relatives and a higher genotyping cost to achieve similar power as population-based methods [3], [4]. The successful recruitment of families is often difficult, especially for late onset disease. As a result, the majority of GWAS are conducted using a population-based design. The second strategy leverages the fact that in the context of GWAS, the vast majority of the SNPs are not associated with the trait under study and therefore can be used to infer ancestry and evaluate/adjust for PS. One popular type of methods, e.g. EIGENSTRAT, construct principle components (PCs) on the data and infer a continuous axis of genetic disparity [5]. Since this method employees the study data ifself to construct the eigenvector space, we term it as “self-eigen” method. Afterwards, the GWAS tests are corrected by adjusting simultaneously for top-ranked PCs, where the number of PCs can be determined either analytically [5], [6] or by permutation [7]. Based on high density SNP array data, the self-eigen approaches demonstrated excellent sensitivity. For example, substructure within the European population was resolved using ∼40,000 random markers [8]. High-density SNP genotyping has also elucidated the substructure in the Finnish population and even individual ancestry at a very high resolution [9].

## Results

As a drawback, self-eigen does not directly infer origin and magnitude of the PS, where such information is important, especially for populations of recent admixture. Therefore, we developed a new eigenvector based method (termed “hgdp-eigen”) to overcome this challenge. It constructs the eigenvector space using the Human Genetics Diversity Panel (HGDP) [10], and then projects the study cohort onto this space (**Methods**). The hgdp-eigen consists two steps. The first step is actually running self-eigen on the HGDP dataset, where our results were identical to previous reports on this set [10] (Figure S1). Briefly, the first four PC dimensions clearly separated populations with respect to the major continents, and beginning with the the 5^th^ dimension we were able to see the finer separation among populations in Africa. In the second step, we projected genotype of study subjects to the PC space built in step 1 and derive the subjects' coordinates on each PC dimension.

To assess the hgdp-eigen, we assembled a human liver-specific cohort (HLC) comprised of over 1,000 individuals, broadly representing three ethnic groups: Caucasian American (CA), Hispanic American (HA), and African American (AA). DNA and RNA were isolated from each of the liver samples, and genotype data for each DNA sample was generated using the Illumina 650Y genotyping array and expression data for each RNA sample was generated using a whole-genome custom Agilent gene expression array. To minimize the effects of assay artifacts, we applied very stringent data quality filters [11]. We then applied both the hgdp-eigen and self-eigen methods to the HLC genotype data (**Methods**).

**Figure 1:**
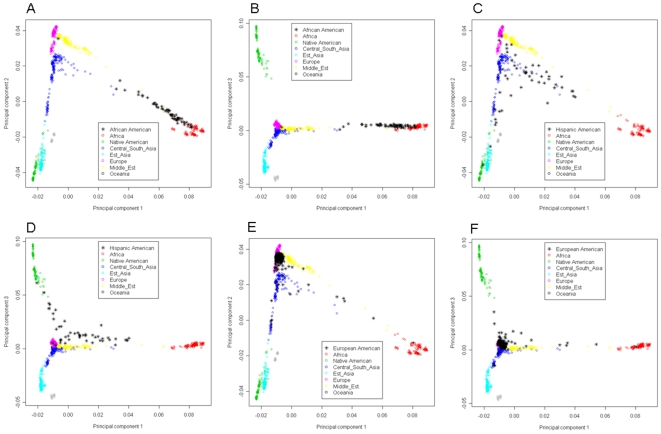
We conducted PCA on the HGDP dataset and observed consistent results as Li et al [10]. The HGDP-PC space can separate world populations with excellent resolution (Figure S1)[10]. Further, we projected the liver study subjects to the HGDP-PC space. A and B, African American subjects; C and D, Hispanic Americans; E and F, European Americans.

The hgdp-eigen took advantage of the HGDP PC space by providing us with a global context of geographically defined world populations (Figure 1). In this space the AA group in the HLC was spread continuously between HGDP African and European samples, although the center was closer to HGDP-African. Leveraging HGDP reference populations also allowed us to quantify the magnitude of PS. Three AA subjects were roughly equal distance from the HGDP-European and HGDP-African clusters, suggesting their ancestry component is half-European and half-African. One AA subject was located in the HGDP-European cluster, suggesting a possible classification error at or after the time the sample was collected. From the 2^nd^ and 3^rd^ HGDP-PC dimensions, we concluded there were no Asian or Native American ancestries represented in AA (Figures 1A&B). The PS in HA subjects were also well-detected (Figures 1C&D). The HA subjects were located closest to the European cluster, however, also showed substantial Native America and African Ancestries. Although these subjects were mainly on the European-African and European-Native American axes, several were located between these two axes (Figure 1C), suggesting a three-way population admixture (European, Africa and Native American). Moreover, HA did not carry East Asia ancestry (shown in the 3^rd^ HGDP-PC dimension, Figure 1D). In contrast, AA samples formed a fine line along the European-Africa axis (Figures 1A&B). CA were more genetically homogenous compared to AA and HA (Figures 1E&F). Nearly all samples were very close to the HGDP-European cluster, although a number exhibited admixture with the African, Native American and/or East Asian populations. Again, the hgdp-eigen quantified the magnitude of admixture. Interestingly, several self-reported CA subjects show ∼50% African ancestry, indicating considerable genetic admixture in American Caucasian populations. We explored higher HGDP-PC dimensions to further refine the origin of AA subjects from the African continent (Figure S1G). Previous reports indicate that the 5^th^ and 6^th^ dimensions reveal the seven populations collected in Africa (Figure S1E and Figure S1F) [10]; these seven populations each formed tight clusters and were well-separated. The Bantu groups from Kenya and South Africa were closely located in this space [10]. Interestingly, the AA subjects in the HLC fell close the Bantu cluster.

**Figure 2:**
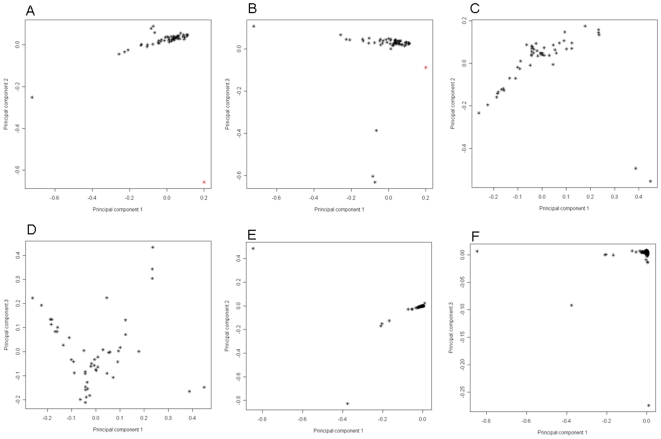
We also applied self-eigen on the HLC, where the PC space was defined by the study sample itself. The African Americans (A and B), Hispanic Americans (C and D) and European Americans (E and F) showed stratification similar but not identical to those in Figure 1.

For comparison, we also applied the self-eigen to the HLC (Figure 2). In the first three PC dimensions, the AA subjects largely formed a continuous line, representing the admixture of African and European ancestries. We identified a few outliers where the origin of ancestry could not be determined (Figures 2A&B, red color). The HA exhibited a V-shaped formation that, aided by the conclusions drawn from Figure 1, we interpret as the admixture of European, African and Native American ancestries (Figures 2C&D). In Figure 2E&F, the CA formed a tight cluster (similar as in Figures 1E&F), although there were a few outliers with unknown origin of ancestry.

Given the acknowledged PS in even relatively homogeneous populations (e.g. European or Finnish), it would be natural to ask about the extent and impact of PS on GWAS in practical settings. More importantly, despite the routine adjustment for PS in GWAS (e.g. using self-eigen), no empirical studies have been carried out to date to assess the impact of these adjustments on statistical power. The large number of phenotypes scored in the HLC provides a path to estimate the impact of PS on GWAS empirically [12], [13], free of assumptions underlying the theoretical arguments and simulation studies. In the HLC, expression quantitative trait locus (eQTL) mapping is a type of GWAS in which the association between gene expression traits and SNP genotypes are tested. Because the structural gene corresponding to the expression trait is always known, we are able to partition the eQTLs identified for any given trait as cis-acting (the structural gene corresponding to the expression trait and the associated SNP are within 1 million base pairs) or trans-acting (the structural gene and the associated SNP are more than 1 million base pairs away or are located on different chromosomes)[11], [14].

Given the considerable population differences observed for gene expression traits [12], the extent of population stratification exhibited in the HLC would likely introduce severe confounding in eQTL mapping. Therefore, we attempted to adjust the eQTL mapping for the subjects' coordinates derived from either the hgdp-eigen or self-eigen method. In total, three different analyses were carried out: (1) unadjusted, (2) self-eigen adjusted, and (3) hgdp-eigen adjusted (Tables 1, 2 and 3). Based on the TW statistics [5], [6], we adjusted the top three eigenvectors in AA and HA, and the top ten eigenvectors in CA. Single-marker Kruskal-Wallis tests were then conducted to identify associations for each trait-SNP pair, and we empirically estimate the false discovery rate (FDR, **Methods**)[13], [15].

**Table 1:** eQTL Mapping in Caucasian American (N = 979).

Adjustment		10% FDR	30% FDR
unadj	cis-eQTL p-value_cutoff_	7.9e-5	3.6e-4
	trans-eQTL p-value_cutoff_	6.0e-9	3.2e-8
	number of cis-eQTLs	7101	10044
	number of trans-eQTLs	607	982
Self - eigen	cis-eQTL p-value_cutoff_	7.4e-5	3.3e-4
	trans-eQTL p-value_cutoff_	5.9e-9	2.6e-8
	number of cis-eQTLs	6958	9647
	number of trans-eQTLs	582	861
Hgdp -eigen	cis-eQTL p-value_cutoff_	8.3e-5	3.6e-4
	trans-eQTL p-value_cutoff_	8.0e-9	2.6e-8
	number of cis-eQTLs	7063	9847
	number of trans-eQTLs	613	836

**Table 2:** eQTL Mapping in African American (N = 59).

Adjustment		10% FDR	30% FDR
unadj	cis-eQTL p-value_cutoff_	9.5e-6	2.9e-5
	trans-eQTL p-value_cutoff_	-	-
	number of cis-eQTLs	65	132
	number of trans-eQTLs	0	0
Self - eigen	cis-eQTL p-value_cutoff_	2.8e-6	4.7e-6
	trans-eQTL p-value_cutoff_	-	-
	number of cis-eQTLs	18	22
	number of trans-eQTLs	0	0
Hgdp -eigen	cis-eQTL p-value_cutoff_	6.2e-6	8.9e-6
	trans-eQTL p-value_cutoff_	-	-
	number of cis-eQTLs	21	30
	number of trans-eQTLs	0	0

**Table 3:** eQTL Mapping in Hispanic American (N = 49).

Adjustment		10% FDR	30% FDR
unadj	cis-eQTL p-value_cutoff_	6.1e-6	3.5e-5
	trans-eQTL p-value_cutoff_	1.0e-7	1.0e-7
	number of cis-eQTLs	33	105
	number of trans-eQTLs	1	1
Self - eigen	cis-eQTL p-value_cutoff_	7.9e-6	2.4e-5
	trans-eQTL p-value_cutoff_	-	2.4e-7
	number of cis-eQTLs	21	50
	number of trans-eQTLs	0	3
Hgdp -eigen	cis-eQTL p-value_cutoff_	9.3e-6	2.0e-5
	trans-eQTL p-value_cutoff_	-	-
	number of cis-eQTLs	24	55
	number of trans-eQTLs	0	0

With a sample size of N = 979, we had excellent statistical power to detect cis-eQTLs in the CA (Table 1). At a 10% FDR, we detected 7,101 cis-eQTLs (unadjusted). In other words, expression levels of 7,101 transcripts were significantly affected by DNA variations near the corresponding structural genes. In contrast, the self-eigen or hgdp-eigen adjusted analysis led to a slightly fewer cis-eQTLs, suggesting that the adjustments either resulted in a reduction in statistical power or reduced the number of artifactual eQTL induced by PS. Nevertheless, the results of the three analyses were highly consistent, indicating that PS is not a major confounder in the CA group. Given the significant multiple-testing penalty, we detected far fewer trans-eQTL, suggesting that even larger sample sizes will be necessary to fully characterize the trans-eQTL architecture in the liver tissue. The unadjusted and eigenvector-adjusted analyses led to consistent trans-eQTL detection results.

Due to the modest sample sizes in the AA and HA, we only had statistical power to detect cis eQTLs (Tables 2 and 3). In contrast to findings for the CA, both self-eigen and hgdp-eigen adjustments greatly reduced the number of cis-eQTL in AA and HA. At a 10% FDR, the unadjusted analysis revealed 65 cis-eQTLs in AA. However, we only found 18 and 21 cis-eQTLs in the self-eigen and hgdp-eigen adjusted analyses, respectively. Again, the reduced number of eQTLs could result from (1) the adjustment diminishing the statistical power, therefore, missing true positives, or (2) the adjustment methods removing the false findings caused by PS. We assessed these two possibilities via a number of paths. First, we examined the consistency of the cis-eQTLs detected in the three ethnic groups (Table S1 and Table S2). Strikingly, the majority of the AA and HA cis-eQTLs (unadjusted analysis) also existed in CA. Herein, we only looked at the self-eigen adjusted CA results in order to rule out possible PS confounding in the CA set. Of the 65 AA cis-eQTLs detected at a 10% FDR, 55 were also detected in the CA. This represents a highly significant overlap (Fisher Exact Test p-value = 1.45E-33). These confirmed eQTLs were actually all very strong (p-value<1E-20, Table S1 and Table S2) in the CA. Similarly, 29 of the 33 HA cis-eQTLs were also detected in the CA. At a 10% FDR, we would expect about 6 and 3 false cis-eQTLs by random chance in the AA and HA, respectively. Therefore, nearly all of the cis-eQTLs identified in the AA and HA groups were confirmed in CA, suggesting they are not PS artifacts.

In addition, we looked at the effect size of the eQTLs (10% FDR). Because the test statistic for the Kruskal-Wallis test does not reflect the effect size, we used the r^2^ estimate from the robust linear model, Trait_adj_ ∼ genotype, to estimate effect sizes. (Here Trait_adj_ denotes the gene expression value already adjusted for age and gender.) Among AA eQTLs, the mean, median and standard deviation of r^2^ were 0.43, 0.46 and 0.14, respectively. Among HA eQTLs, the mean, median and standard deviation of r^2^ were 0.54, 0.56 and 0.16, respectively. And for CA eQTLs that are confirmed in AA or HA, the mean, median and standard deviation of r^2^ are 0.52, 0.54 and 0.19, respectively.

Further, we investigated whether the adjustment reduced statistical power, leading to a failure to detect many true cis eQTL that would have been found without the adjustment. The large number of phenotypes (∼40,000 expression traits) provided a path to empirically estimate power (**Methods**) [13]. Following this rationale, we conducted eQTL mappings with adjustment for the top 1 or 2 eigenvectors, and compared statistical power (i.e., number of cis-eQTLs) in Table S3 and Table S4. Interestingly, the statistical power generally followed the pattern: unadjusted count > adjusted for the top 1 eigenvector count > adjusted for the top 2 eigenvectors count > adjusted for the top 3 eigenvectors count.

Finally, if we assume that many of the cis eQTLs in the AA and HA were caused by PS and consequently excluded by eigenvector adjustment, the false trait-SNP associations should bear the following properties: (1) the gene expression trait should be differentially expressed between subpopulations, and (2) the SNP allele frequency should be different between the subpopulations. In fact, for any gene whose expression varied significantly between subpopulations and any SNP whose frequency also varied significantly between subpopulations, we would detect associations for such trait-SNP pair. Because SNPs with different allele frequencies among subpopulations should uniformly distribute throughout the genome, we would expect to see the same number of cis-eQTL detected no matter where we decided to place the 2 Mbp window defining the cis region of interest. That is, if we randomly chose 2 Mbp windows for each gene expression trait and counted the number of pseudo cis-eQTL detected, we would expect it to be close to the number of true cis eQTL. We performed this simulation in the HLC for the AA and HA, randomly placing the 2 Mbp window for each gene and conduct cis-eQTL mapping for over 1000 runs. We found an average of 9.92 and 2.03 pseudo cis-eQTLs in AA and HA, respectively, significantly less than the 65 and 33 true cis-eQTL. Therefore, we are able to reject our hypothesis that the cis-eQTL detected in the AA and HA were mostly driven by PS and support of the hypothesis that the majority of cis-eQTLs in the unadjusted analysis were real.

To illustrate the impact adjusting for PS can have on identifying disease susceptibility loci, we intersected the HLC eQTLs with the set of SNPs in the public GWAS databases identified and replicated as associated with common human disease [16]. We and others have demonstrated that eQTLs are a powerful tool for interpreting pathways underlying GWAS hits and extend our understanding for SNP-disease associations [11], [17], [18]. In the AA unadjusted analysis, we identified the SNP rs646776 as significantly associated with SORT1 and PSRC1 liver expression. These results were also confirmed in the CA. This SNP has been shown to associate with lipid levels and coronary heart disease risk [18]–[20]. There are many genes in the region of the rs646776 locus (Figure S3), and CELSR2, PSRC1 and SORT1 genes have been suggested to mediate the function of this SNP (or functional SNPs in LD)[11], [18]–[20]. However, self-eigen or hgdp-eigen adjusted analysis only found the rs646776-SORT1 eQTL, but missed the rs646776-PSRC1 association. In addition, the SNP rs10903129 has been associated with lipid levels and coronary heart disease risk and the gene TMEM57 has been implicated as the main candidate susceptibility gene in this locus[21]. Again, using the 59 AA subjects, the unadjusted analysis revealed the rs10903129-TMEM57 eQTL, but not in the adjusted analysis.

## Discussion

In this study, we have analyzed three major ethnic groups in the United States, Caucasian American, African American and Hispanic American. Hgdp-eigen methods provided valuable information on the origin of the admixtures. For example, it revealed the African and Native American components in the HA genome. Second, HGDP-PC quantified the magnitude of the PS. In contrast, the self-eigen method simply detected the PS without inferring the origin of the genetic flow and the magnitude. Lastly, the HGDP-PC space was constructed on the HDGP sample, capturing the primary allele frequent differences of world populations, robust to the study cohorts. Comparing Figures 1C and 2C, we found the CA subjects were better separated out in HGDP-PC space. The HDGP-PC approach was robust in capturing the African, East Asian, and Native American components in the CA genomes, however, the self-eigen method was heavily influenced by a few outliers. For sample, the CA subjects showed a clear Y-formation in HGDP-eigen space (Figure 1F), reflecting the European-African, and European-Asian and European-Native American admixtures. We can also identify a few subjects midway between the HGDP-European and HGDP-African clustered, suggesting they have roughly an equal dosage of European and African ancestry. In contrast, the CA subjects formed a tight cluster in the self-eigen space (Figure 2F), with a few outliers spreading out. The 3^rd^ dimension is primarily defined by two outliers (Figure 2F) with unknown ancestry. On the other hand, although the HGDP-eigen approach is very useful in separating and visualizing the population substructures, it may not be the most appropriate method for adjusting for PS in association tests. Because HGDP-eigen includes many eigenvectors that are irrelevant for a particular study cohort (e.g. East Asian in our present study), use of these eigenvectors in a cohort such as ours may “correct” for biases that are not present. The self-eigen approach would be adequate in adjusting PS in association tests.

Assessing the extent of PS confounding is an important but challenging task. There were attempts to address this issue using simulation and small empirical datasets (e.g. the lactase gene) [6]. However, the simulations were based on assumptions that might not be true in practice, and the empirical data points were too few to draw meaningful statistical inferences [6]. Here, we relied on ∼40,000 expression traits. Many such traits showed different expression levels in various distributions [12], therefore, the eQTL mapping were subject to PS confounding. Interestingly, although PS was clearly detected in our liver study CA subjects, it did not lead to a severe bias. The eigenvector-adjusted eQTLs were consistent with the naïve results, implying it is generally safe to conduct regular GWAS tests in cohorts of European ancestry. Further, while PS is a bigger issue in AA and HA, most of the discoveries we identified are likely to be real since they were confirmed in the Caucasian cohort. In addition, simulation showed there should be an average of 9.92 and 2.03 false cis-eQTLs in AA and HA, respectively, many fewer than the total number of positives we identified. Due to the small sample size of AA and HA, we are capable of capturing only the strongest signals, which seem to be real given the supporting evidence from multiple sources. Such results suggest that even for GWAS on AA and HA population, the strong findings are not likely to be a PS artifact. In any case, larger sample sizes are necessary to examine whether weaker associations found in admixture population are more vulnerable to PS.

In contrast, the eigenvector-adjustment greatly reduced the number of cis-eQTL findings in AA and HA, due to a loss of statistical power. The underlying rationale is easy to understand. Many SNPs showed considerable allele frequency differences (e.g. ≥0.1) among ethnic groups. In our data, 60.1% SNPs showed ≥0.1 allele frequency difference between AA and CA (Figure S2). If the true causal SNPs also had such characteristics, within AA subjects, they would have different allele frequencies between (1) subgroups with more African genetic component and (2) subgroups with more European genetic component. Correspondingly, the expression trait value would also be different between the two subgroups. In the unadjusted analysis, this eQTL could be identified passing a stringent FDR (e.g. 10%). However, the eigenvector adjustment removed part of the trait value and allele frequency difference between the two subgroups, and as a result, this eQTL could be missed due to the power reduction. From the statistics viewpoint, the eigenvector adjustment controlled the type I error at the cost of the statistical power (inflated the type II error). Such a trade-off is particularly prominent in admixed populations. Alternatively, our results suggest that because the bias introduced by PS may not be as significant as once feared and that performing genome-wide association studies in admixed populations may be a reasonably strategy in increasing samples sizes to maximize power to detect associations. Although, until the impact of PS on GWAS is fully understood, it may be prudent to replicate associations identified from such studies in independent homogenous populations to protect against PS-induced associations.

## Methods

### Liver tissue samples

Liver tissue samples were collected from “Liver Study subjects”, whose detailed characteristics were reported in a separate article [11], [22]. The sample collection was a joint effort of three independent institutes, Vanderbilt University, the University of Pittsburgh, Massachusetts General Hospital and Merck Research Laboratories. About half the subjects are healthy individuals and half are obese patients. The “Liver study” is a retrospective study, and self-reported ethnicity information is gathered in the interview questionnaire. All samples came from individuals who provided written informed consent to make their samples available for scientific research. In addition, all of the samples and patient data were approved for use in this study by IRBs specific to each of the participating organizations. DNA specimens were extracted and genotyped on the Ilmn650Y array. Additionally, we purified RNA from the tissue samples and measured the approximately 40,000 gene transcription levels using the Agilent platform. In total, we have successfully mRNA profiled and SNP genotyped 979, 59 and 49 self-reported Caucasian, African and Hispanic Americans, respectively. Furthermore, we filtered out SNPs with call rate <90%, and totally 574K autosomal SNPs were used in the analysis.

Since the liver tissues [11], [22] were collected by two separate efforts and gene expression profiling was carried out at different times for each group, we normalized the two expression datasets at the gene level to avoid systematic bias. In brief, for every reporter, we applied quantile normalization (implemented in the Affy library of the R statistical package) and forced the trait distribution to be identical between the two tissue sets. We compared the expression levels for AA, HA and CA (randomly selected N = 59 CA samples) using a pair-wise t-test. For AA vs. CA, HA vs. CA, and HA vs. AA, 1.7%, 1.2%, and 0.4% of the genes, respectively, were detected as differentially expressed at the 0.01 level. We note that with such a small sample size (N = 49 HA subjects), the t-test may have modest statistical power to identify differences.

### Human Genetics Diversity Project (HGDP)

938 unrelated individuals from 51 populations (collected in Europe, Middle East, Central/South Asia, Africa, East Asia, America and Oceania) of the HGDP were successfully genotyped using Ilmn650Y [10], and data has been made available to the public. Principal components (PCs) built on over 600K assayed SNPs provide high resolution to separate subjects from different continents. We implemented the EIGENSTRAT algorithm [5], and derived identical results as Li et al [10]. Further, we projected the deLiver subjects to PC space defined by HGDP data (termed as HGDP-PC space) and examined population admixture in our samples. The Caucasian Americans clustered tightly and collocated with HGDP Europeans. However, the eight African Americans show certain degree of admixture, in another word, deviation from the HGDP African populations towards the HGDP European cluster. Such results suggest European genetic components in African American samples.

#### Principal component analysis

To avoid artifacts due to linkage disequilibrium we thin the data by excluding highly correlated SNPs. Then we conducted two versions of PCA. First, the standard methods as implemented in the Eigensoft package [23]. The liver dataset was used to create the PC space, and the subjects' coordinates in every dimension of this space were recorded. In the second PCA version, we constructed the PC space using the HGDP data (termed as HGDP-PC space), and then projected the liver study subjects to this space and derived the coordinates for each dimension. We also computed the Tracy Widom (TW) statistics, which could determine the number of PCs to be adjusted for [5], [6]. In AA and HA, the top three TW statistics were positive, as results, we adjusted the first three PCs in GWAS. Because of the large sample size (N = 979) of CA, many TW statistics were positive, and we adjusted for the first ten PCs in analysis.

#### Association testing

Kruskal-Wallis (KW) one-way analysis of variance was employed in testing association between gene expression traits and genotypes. The KW test can be considered as the non-parametric counterpart to ANOVA for testing equality among groups (e.g., the three genotype groups corresponding to a given SNP). This test does not assume the traits are normally distribute and therefore is more robust to outliers and violations of other assumptions important for successful application of parametric tests. In brief, the KW test was applied on a given trait-SNP pair by first ranking all trait values regardless of genotype, assigning tied values the average of the ranks they would have received had they not been tied. Then we computed the test statistic (K) as
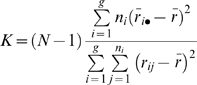
where n_i_ is the number of subjects for genotype i; r_ij_ is the rank of subject j who carried genotype i; N is the entire sample size; and g denotes the number of genotype groups (either 2 or 3 for the groups tested). Finally, the p value was derived using the approximation Pr(χ^2^_g-1_≥K). Before testing the gene expression traits, we adjusted them for age, gender and PCs. This adjustment was carried out by fitting a robust linear model (using the rlm function in the R statistical software package) to each of the gene expression traits,
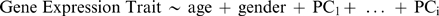
where we adjusted up to three of the most informative PCs. Afterwards, the residual of the linear model was used as input into the Kruskal-Wallis test.

#### Empirical Estimation of FDR

We repeated the eQTL mapping analyses on permuted gene expression data sets to empirically estimate FDR. In each permutation run, we first randomized the patient IDs in the expression file, breaking any association between expression traits and genotypes, while leaving the respective correlation structures among gene expression traits and SNP genotypes intact. Then we repeated the association tests for every expression trait and genotype pair in the permuted sets, leading to a set of null statistics for each permutation. A standard FDR estimator was then applied to the resulting association statistics, as previously carried out on observed and permutation null statistics [15].

#### Empirically estimation of statistical power using large number of phenotypes

Although we could not determine whether a particular discovery was true or false, at a given FDR (e.g. 10%) we knew the proportion (e.g. 90%) of discoveries that were true. Therefore, at a fixed FDR, when two methods resulted in a different number of discoveries (termed as N_1_ and N_2_) there would be (1-FDR)*N_1_ and (1-FDR)*N_2_ true findings, where N_1_/N_2_ is then proportional to the relative power of the two methods.

## Supporting Information

Table S1 Cis eQTL detected in the African American samples from the human liver-specific cohort.
(0.03 MB XLS)

Table S2 Cis eQTL detected in the Hispanic American samples from the human liver-specific cohort.
(0.03 MB XLS)

Table S3 eQTL Mapping in African American, adjusted for the top 1 or 2 eigenvectors. Fifty-five of the 65 AA cis-eQTLs (detected using unadjusted traits at 10% FDR) also exist as CA cis-eQTLs (detected using self-eigen adjusted traits at 10% FDR), with an enrichment pvalue = 1.45E-33.
(0.05 MB DOC)

Table S4 eQTL Mapping in Hispanic American, adjusted for the top 1 or 2 eigenvectors. Twenty-nine of the 33 HA cis-eQTLs (detected using unadjusted traits at 10% FDR) also exist as CA cis-eQTLs (detected using self-eigen adjusted traits at 10% FDR), with an enrichment pvalue = 4.87E-20.
(0.04 MB DOC)

Figure S1 We conducted PCA on the HGDP dataset and observed consistent results as Li et al. A, the 1st PC separates Africa vs. Non-Africa populations and the 2nd PC separates East Asia, Native America and Oceania from other Non-Africa populations; B, the 3rd PC separates Native America from other populations; C, the 4th component separates Oceania from others; D, the 5th component separates different populations in Africa; E, the 6th component continues to separate different populations in Africa; F, in the space formed by the 5th and the 6th HGDP-PCs, African populations of various culture/language/locations were well separated; G, we projected the AA subjects to the 5th and 6th HGPD PCs, interestingly, the AA samples were located very close to Bantu groups.
(9.23 MB TIF)

Figure S2 We compared the allele frequency among the three ethnic groups of the liver study (A, African America vs. Caucasian American; B, African America vs. Hispanic American; and C, Caucasian American vs. Hispanic American). A considerable percentage of SNPs showed large allele frequency disparities (e.g., < = 0.1). Further, we applied simple Chi-square test and found many of the differences were significant (D, African America vs. Caucasian American; E, African America vs. Hispanic American; and F, Caucasian American vs. Hispanic American).
(8.82 MB TIF)

Figure S3 There are many genes near the SNP rs646776 locus, including PSRC1 and SORT1.
(1.80 MB TIF)
