# Supplementary material for: Magnitude of Stratification in Human Populations and Impacts on Genome Wide Association Studies
Source: PLoS One. 2010 Jan 13;5(1):e8695. doi: 10.1371/journal.pone.0008695 (PMC2805717; doi:10.1371/journal.pone.0008695)
Supplement: Table S3 — eQTL Mapping in African American, adjusted for the top 1 or 2 eigenvectors. Fifty-five of the 65 AA cis-eQTLs (detected using unadjusted traits at 10% FDR) also exist as CA cis-eQTLs (detected using self-eigen adjusted traits at 10% FDR), with an enrichment pvalue = 1.45E-33. (0.05 MB DOC) [file pone.0008695.s003.doc]

Table S3

| **Adjustment** |  |  |  | 10% FDR | 30% FDR |  |
| --- | --- | --- | --- | --- | --- | --- |
| unadj | cis-eQTL p-value_cutoff_ |  |  | 9.5e-6 | 2.9e-5 |  |
|  | trans-eQTL p-value_cutoff_ |  |  | - | - |  |
|  | number of cis-eQTLs |  |  | 65 | 132 |  |
|  | number of trans-eQTLs |  |  | 0 | 0 |  |
| Top 1 eigenvector | cis-eQTL p-value_cutoff_ |  |  | 6.6e-6 | 1.1e-5 |  |
|  | trans-eQTL p-value_cutoff_ |  |  | - | - |  |
|  | number of cis-eQTLs |  |  | 53 | 66 |  |
|  | number of trans-eQTLs |  |  | 0 | 0 |  |
| Top 2 eigenvectors | cis-eQTL p-value_cutoff_ |  |  | 4.9e-6 | 1.8e-5 |  |
|  | trans-eQTL p-value_cutoff_ |  |  | - | - |  |
|  | number of cis-eQTLs |  |  | 28 | 72 |  |
|  | number of trans-eQTLs |  |  | 0 | 0 |  |
