# Supplementary material for: Magnitude of Stratification in Human Populations and Impacts on Genome Wide Association Studies
Source: PLoS One. 2010 Jan 13;5(1):e8695. doi: 10.1371/journal.pone.0008695 (PMC2805717; doi:10.1371/journal.pone.0008695)
Supplement: Table S4 — eQTL Mapping in Hispanic American, adjusted for the top 1 or 2 eigenvectors. Twenty-nine of the 33 HA cis-eQTLs (detected using unadjusted traits at 10% FDR) also exist as CA cis-eQTLs (detected using self-eigen adjusted traits at 10% FDR), with an enrichment pvalue = 4.87E-20. (0.04 MB DOC) [file pone.0008695.s004.doc]

Table S4

| **Adjustment** |  |  |  | 10% FDR | 30% FDR |  |
| --- | --- | --- | --- | --- | --- | --- |
| unadj | cis-eQTL p-value_cutoff_ |  |  | 6.1e-6 | 3.5e-5 |  |
|  | trans-eQTL p-value_cutoff_ |  |  | 1.0e-7 | 1.0e-7 |  |
|  | number of cis-eQTLs |  |  | 33 | 105 |  |
|  | number of trans-eQTLs |  |  | 1 | 1 |  |
| Top 1 eigenvector | cis-eQTL p-value_cutoff_ |  |  | 1.4e-5 | 3.1e-5 |  |
|  | trans-eQTL p-value_cutoff_ |  |  | 1.4e-7 | 1.4e-7 |  |
|  | number of cis-eQTLs |  |  | 41 | 77 |  |
|  | number of trans-eQTLs |  |  | 1 | 1 |  |
| Top 2 eigenvectors | cis-eQTL p-value_cutoff_ |  |  | 6.6e-6 | 2.0e-5 |  |
|  | trans-eQTL p-value_cutoff_ |  |  | - | - |  |
|  | number of cis-eQTLs |  |  | 18 | 49 |  |
|  | number of trans-eQTLs |  |  | 0 | 0 |  |
