# Supplementary figures and images for: Magnitude of Stratification in Human Populations and Impacts on Genome Wide Association Studies
Source: PLoS One. 2010 Jan 13;5(1):e8695. doi: 10.1371/journal.pone.0008695 (PMC2805717; doi:10.1371/journal.pone.0008695)

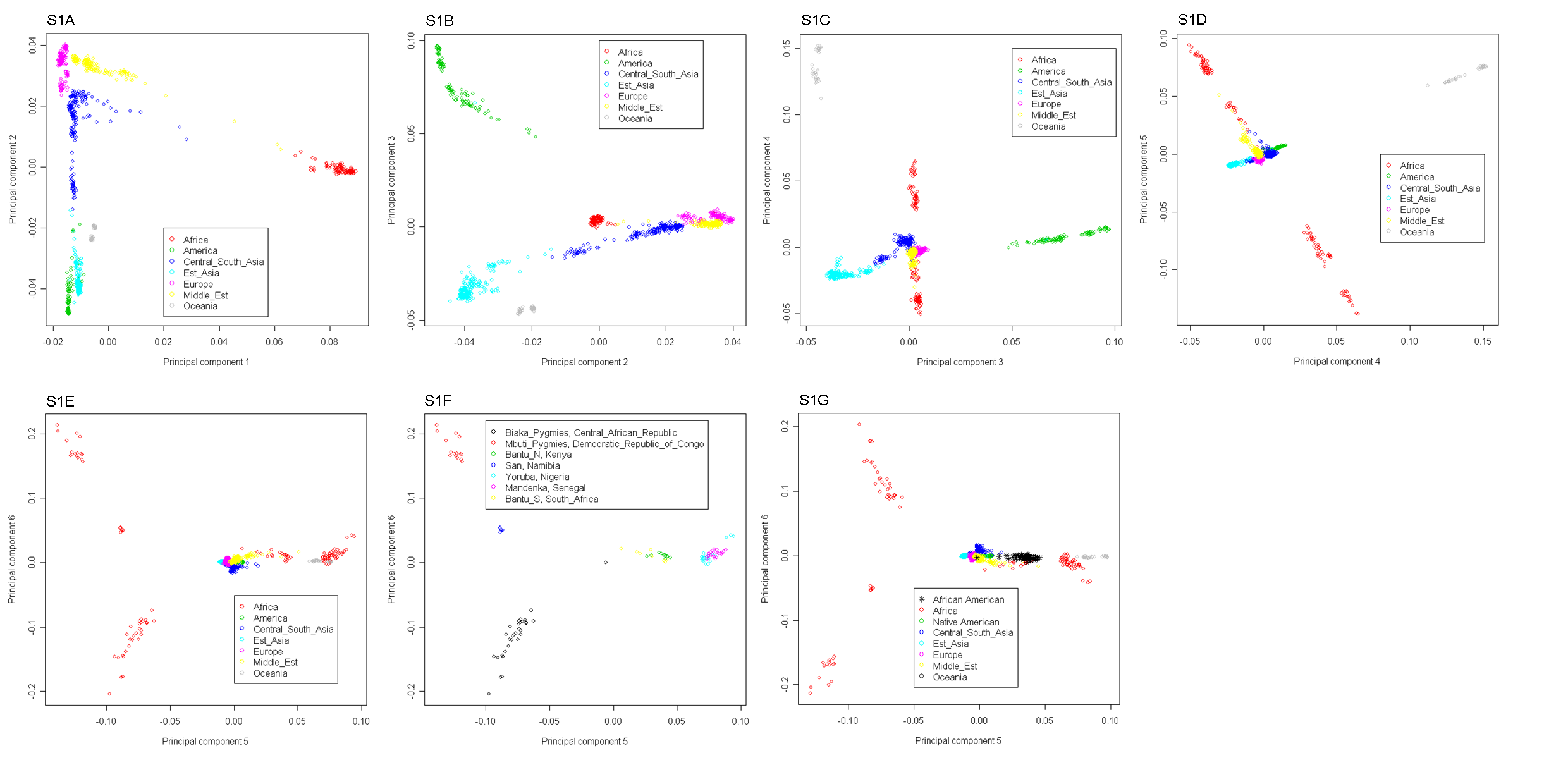

Supplement: Figure S1 — We conducted PCA on the HGDP dataset and observed consistent results as Li et al. A, the 1st PC separates Africa vs. Non-Africa populations and the 2nd PC separates East Asia, Native America and Oceania from other Non-Africa populations; B, the 3rd PC separates Native America from other populations; C, the 4th component separates Oceania from others; D, the 5th component separates different populations in Africa; E, the 6th component continues to separate different populations in Africa; F, in the space formed by the 5th and the 6th HGDP-PCs, African populations of various culture/language/locations were well separated; G, we projected the AA subjects to the 5th and 6th HGPD PCs, interestingly, the AA samples were located very close to Bantu groups. (9.23 MB TIF) [file pone.0008695.s005.tif]

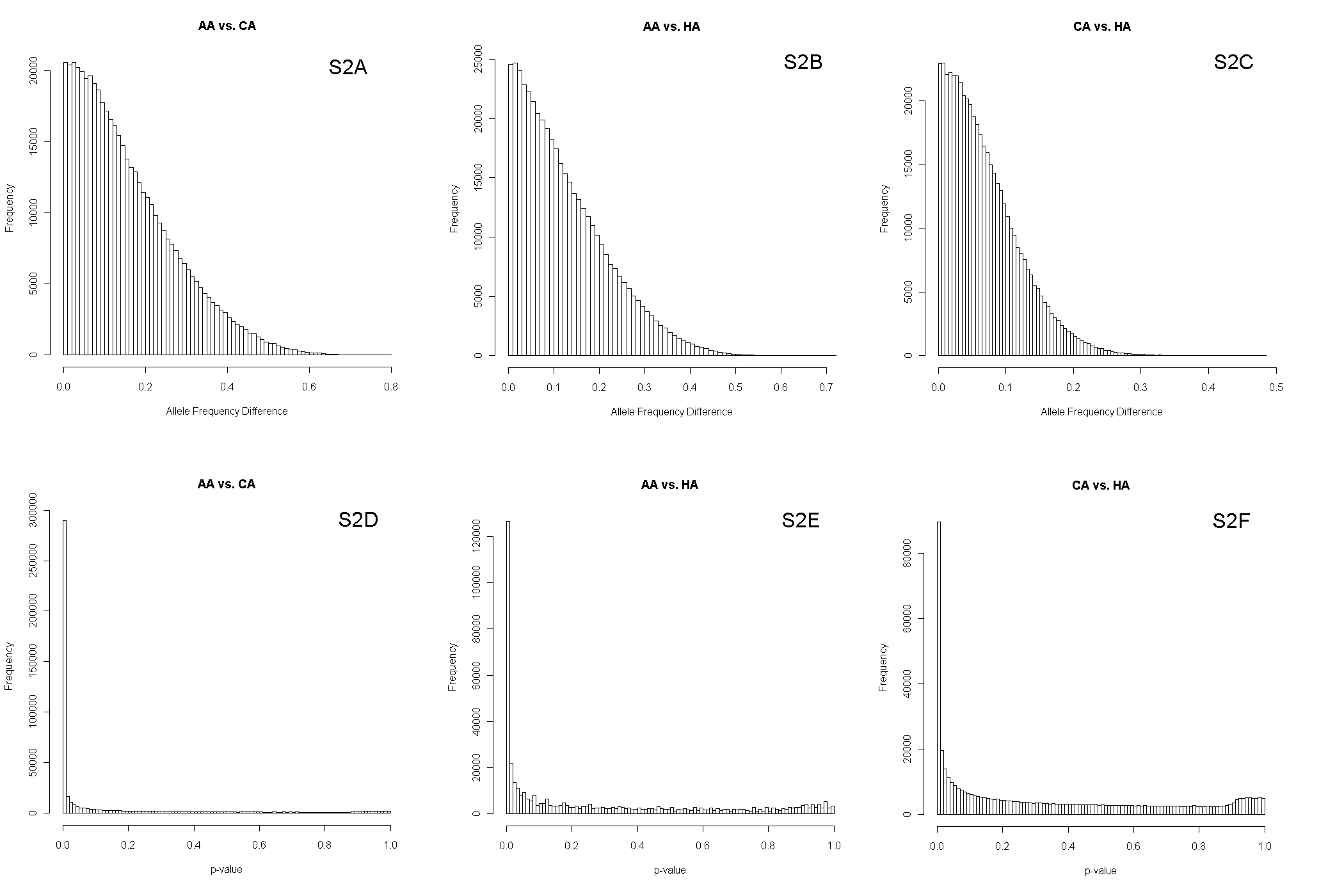

Supplement: Figure S2 — We compared the allele frequency among the three ethnic groups of the liver study (A, African America vs. Caucasian American; B, African America vs. Hispanic American; and C, Caucasian American vs. Hispanic American). A considerable percentage of SNPs showed large allele frequency disparities (e.g., < = 0.1). Further, we applied simple Chi-square test and found many of the differences were significant (D, African America vs. Caucasian American; E, African America vs. Hispanic American; and F, Caucasian American vs. Hispanic American). (8.82 MB TIF) [file pone.0008695.s006.tif]

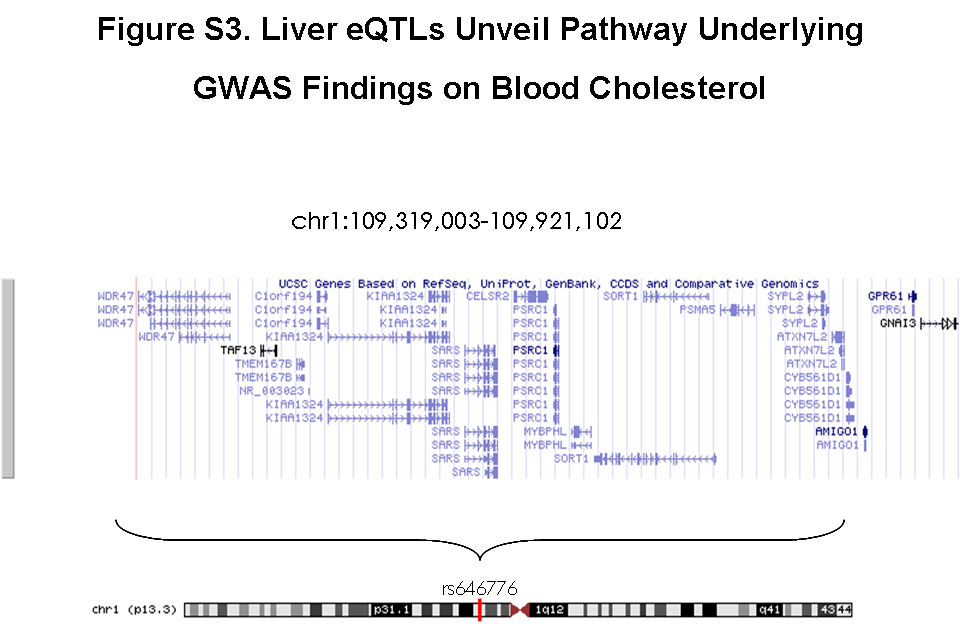

Supplement: Figure S3 — There are many genes near the SNP rs646776 locus, including PSRC1 and SORT1. (1.80 MB TIF) [file pone.0008695.s007.tif]
